# Tensor description of X-ray magnetic dichroism at the Fe *L*_2,3_-edges of Fe_3_O_4_

**DOI:** 10.1107/S1600577520015027

**Published:** 2021-01-01

**Authors:** Hebatalla Elnaggar, Maurits W. Haverkort, Mai Hussein Hamed, Sarnjeet S. Dhesi, Frank M. F. de Groot

**Affiliations:** a Debye Institute for Nanomaterials Science, Utrecht University, 99 Universiteitsweg, Utrecht 3584 CG, The Netherlands; b Institute of Theoretical Physics, Heidelberg University, 19 Philosophenweg, Heidelberg 69120, Germany; c Jülich Centre for Neutron Science, Forschungszentrum Juelich GmbH, Jülich 52425, Germany; d Faculty of Science, Helwan University, Cairo 11795, Egypt; e Diamond Light Source, Harwell Science and Innovation Campus, Didcot OC11 0DE, United Kingdom

**Keywords:** X-ray absorption spectroscopy, magnetic dichroism, fundamental spectra, tensor analysis

## Abstract

A procedure to build the optical conductivity tensor that describes the full magneto-optical response of the system from experimental measurements is presented. Applied to the Fe *L*_2,3_-edge of a 38.85 nm Fe_3_O_4_/SrTiO_3_ (001) thin-film, it is shown that the computed polarization dependence using the conductivity tensor is in excellent agreement with that experimentally measured. Furthermore, the magnetic field angular dependence is discussed using a set of fundamental spectra expanded on spherical harmonics. It is shown that the convergence of this expansion depends on the details of the ground state of the system in question and in particular on the valence-state spin–orbit coupling. While a cubic expansion up to the third order explains the angular-dependent X-ray magnetic linear dichroism of Fe^3+^ well, higher-order terms are required for Fe^2+^ when the orbital moment is not quenched.

## Abstract 2

A general description of magnetic dichroism effects in X-ray absorption spectroscopy using a few fundamental spectra extracted from experimental measurements is provided. The accuracy of the procedure is investigated theoretically.

## 1. Introduction   

The determination of the electronic and magnetic structure of engineered magnetic nanostructures is essential to tailor their properties for technological applications such as information storage, spin transport and sensing technology. These devices often rely on magnetic thin-films and nanostructures comprising multiple layers, such as for instance transition metal-oxides magnetic tunnel junctions and exchange biased systems. X-ray magnetic dichroism spectroscopy is a powerful tool that can provide element- and site-specific magnetic information in heteromagnetic nanostructures (Kuiper *et al.*, 1993 ▸; Nunez Regueiro *et al.*, 1995 ▸; Alders *et al.*, 1998 ▸; Scholl *et al.*, 2000 ▸; Hillebrecht *et al.*, 2001 ▸; Haverkort *et al.*, 2004 ▸; van der Laan, 2013 ▸; Luo *et al.*, 2019 ▸). X-ray magnetic circular dichroism (XMCD) can be used to determine the spin and orbital magnetic moments using sum rules (Carra *et al.*, 1993 ▸) while X-ray magnetic linear dichroism (XMLD) can be used to determine the site symmetry, anisotropic magnetic moments and spin–orbit interaction (Lüning *et al.*, 2003 ▸; Csiszar *et al.*, 2005 ▸; Arenholz *et al.*, 2006 ▸; Finazzi *et al.*, 2006 ▸; van der Laan *et al.*, 2011 ▸; Chen *et al.*, 1992 ▸, 2010 ▸; Iga *et al.*, 2004 ▸). However, using dichroism experiments for magnetometry is far from being straightforward because it requires an understanding of the spectral shape and magnitude of the dichroism signal as well as its dependence on the relative orientation of the X-ray polarization, the exchange field and the crystallographic axes.

The aim of this work is to provide a general method to construct and analyse dichroism effects in dipole transitions such as at the Fe *L*_2,3_-edge in magnetite (Fe_3_O_4_). We illustrate the procedure to build the conductivity tensor from a few well chosen experimental measurements describing all possible dichroism effects at a single magnetic field orientation. Furthermore, the angular dependence of the magnetic field is discussed using a set of fundamental spectra expanded using spherical harmonics which can describe the full magneto-optical response of the system (Haverkort *et al.*, 2010 ▸). Such expansions have been used previously to explain the angular dependence of XMLD (Arenholz *et al.*, 2006 ▸, 2007 ▸; van der Laan *et al.*, 2008 ▸, 2011 ▸), yet the new aspect we provide in this work is a thorough inspection of the convergence of the expansion using a comprehensive set of XMLD data measured on Fe_3_O_4_ in combination with theoretical calculations. Fe_3_O_4_ serves as an adequate model system: it is a ferrimagnetic mixed-valence strongly correlated system containing two different Fe sites where Fe^3+^ ions reside in tetrahedral (*T*_*d*_) coordinated intersites (*A* sites), while both Fe^2+^ and Fe^3+^ ions are in octahedral (*O*_*h*_) coordinated intersites (*B* sites). This provides us with an opportunity to study the effect of the electronic structure on the quality of the expansion between the orbital singlet Fe^3+^ and the orbital triplet Fe^2+^ ions.

## 2. Methods   

### 2.1. Experimental   

The Fe_3_O_4_ thin-film was grown on a conductive 0.1% Nb-doped SrTiO_3_ (001) TiO_2_-terminated substrate using pulsed laser deposition as reported by Hamed *et al.* (2019 ▸). The film thickness and surface roughness were concluded to be 38.85 nm and 0.4 nm, respectively, from X-ray reflectivity measurements (Fig. 10 of Appendix *A* [App appa]). The Verwey transition was observed at 114.97 ± 0.29 K from the magnetization versus temperature measurements in zero field cooling mode with 500 Oe applied field (Fig. 11 of Appendix *A* [App appa]). Hysteresis measurements were also performed along the [1,0,0] direction to inspect the saturation of the thin-film below and above the Verwey transition (Fig. 11 of Appendix *A* [App appa]). The largest coercivity is observed for the lowest temperature (*H*_c_ = 0.1 T) and an external magnetic field of ∼0.25 T is required to saturate the in-plane magnetization (see Fig. 12 of Appendix *A* [App appa]). On the contrary, the magnetization is not saturated along the [0,0,1] direction with a field of *H* = 2 T as shown by the XMCD measurement shown in (Fig. 13 of Appendix *A* [App appa]).

X-ray absorption spectroscopy (XAS) measurements were carried out on beamline I06 of Diamond Light Source, UK. The beam spot at the sample position was estimated to be ∼200 µm × 100 µm. The polarization of the beam can be controlled using an Apple-II type undulator to produce linear­ly and circularly polarized X-rays. A vector magnet set to 1 T was used to saturate the magnetization to (nearly) any arbitrary direction. All measurements were performed at *T* = 200 K in a normal-incidence configuration, *i.e.* with the incoming beam impinging at an angle of 90° with respect to the sample surface. The energy resolution was estimated to be ∼200 meV full width at half-maximum (FWHM). The measurements were performed in total electron yield mode. All experimental spectra were first normalized to the incident photon flux. The spectra were then fitted using a model consisting of two error functions to take into account the *L*_2,3_-edge jumps. In addition, a set of Gaussian functions were used to fit the multiplet features of the spectra (refer to Appendix *B* [App appb] for more details). The *L*_2,3_-edge jumps were subtracted from the spectra and the spectra were renormalized to the spectral area.

### 2.2. Computational   

The data treatment and the Kramers–Kronig transformation were performed using Python. Crystal field multiplet calculations were performed using the quantum many-body program *Quanty* (Haverkort *et al.*, 2012 ▸). The Hamiltonian we use is of the form
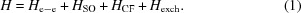
The electron–electron Hamiltonian (*H*_e−e_) is of the form
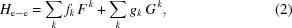
where *F* ^*k*^ (*f*_*k*_) and *G* ^*k*^ (*g*_*k*_) are the Slater–Condon parameter for the radial (angular operators) part of the direct and exchange Coulomb interactions, respectively. The radial integrals are obtained from atomic Hartree–Fock calculation scaled to 70% and 80% for valence and valence-core interactions, respectively, to take into account interatomic screening and mixing effects. This is in line with works in the literature such as those by Arenholz *et al.* (2006 ▸) and Pattrick *et al.* (2002 ▸). This reduction is related to two effects: (i) the 80% reduction is to correct the Hartree–Fock calculations such that they agree with atomic data, as shown by Cowan (1981 ▸) and many others; (ii) the additional reduction to 70% is to take into account the effects of charge transfer; in other words, the nephelauxetic effects.

The spin–orbit Hamiltonian (*H*_SO_) is of the form
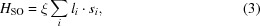
where *l*_*i*_ and *s*_*i*_ are the one electron orbital and spin operators, respectively, and the sum over *i* is over all electrons. The prefactor ξ is an atom-dependent constant (which is to a good approximation material independent) and hence we used here tabulated data for this with ξ = 0.052 eV for 3*d* orbitals and ξ = 8.20 eV for 2*p* orbitals.

The crystal field Hamiltonian (*H*_CF_) is of the form
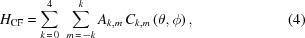
where *C*_*k*,*m*_(θ,ϕ) are the angular crystal field operators expanded on renormalized spherical harmonics and *A*_*k*,*m*_ are proportional to the distortion parameters used in crystal field theory, 10*D*_*q*_, *D*_*s*_ and *D*_*t*_. In cubic symmetry we consider only 10*D*_*q*_ which we found to be 1.25 eV and 0.5 eV for the *B* and *A* sites, respectively, by fitting to the XAS and XMCD spectra. The optimized parameters used for the calculations can be found in Tables 4, 5 and 6. Details of the ground state for the three Fe ions in Fe_3_O_4_ are shown in Appendix *C* [App appc]. We note that we have not taken into account charge transfer effects explicitly in our model. The mixing of iron and oxygen orbitals gives rise to charge transfer effects in core level spectroscopies. It has been shown that neutral experiments on relatively ionic systems map very accurately to the crystal field multiplet model [refer to de Groot & Kotani (2008 ▸) for example]. This is the basis of crystal field theory, where the hybridization is effectively taken care of by the reduction of the Slater integrals from their atomic values, *i.e.* an extra reduction with respect to the 80% reduction of the Hartree–Fock values.

Finally, the magnetic exchange Hamiltonian is given as
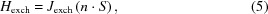
where *S* is the spin operator, *n* is a unit vector giving the direction of the magnetization and *J*_exch_ is the magnitude of the mean-field exchange interaction which we use as 90 meV in our calculation. This value is based on previous 2*p*3*d* RIXS measurements that showed that the spin-flip excitation is observed at this energy [see, for example, Huang *et al.* (2017 ▸) and Elnaggar *et al.* (2019*a* ▸,*b* ▸)].

## 3. Results and discussion   

### 3.1. Construction of the conductivity tensor   

The general XAS cross-section can be expressed by equation (6) where ε is the polarization vector, Im is the imaginary part of the equation and σ is the conductivity tensor describing the material properties (Haverkort *et al.*, 2010 ▸),
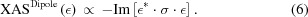
The conductivity tensor is a 3 × 3 matrix for a dipole transition as shown in equation (7). The matrix elements of the conductivity tensor are defined in equation (8) where ψ is the ground state wavefunction, *T*_*x*(*y*)_ = ε_*x*(*y*)_·*r*_*x*(*y*)_ is the dipole transition operator, *H* is the Hamiltonian (taking into account the core-hole effect) and γ is the Lorentzian broadening given by the core-hole lifetime [*L*_3_ = 200 meV and *L*_2_ = 500 meV half width at half-maximum (HWHM) (de Groot, 2005 ▸); *L*_2_ has a larger lifetime broadening due to the Coster–Kronig Auger decay],
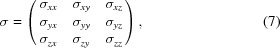


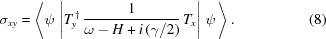
In the most general case, nine independent measurements are required to fully reconstruct the conductivity tensor. However, the crystal symmetry can simplify the conductivity tensor by dictating the equivalence between matrix elements or cancelling out some of the matrix elements. For a cubic crystal system with the magnetic field aligned parallel to the high symmetry [1,0,0] direction, only five of the nine matrix elements are non-zero (three diagonal elements: σ_*xx*_, σ_*yy*_, σ_*zz*_; and two off-diagonal elements: σ_*yz*_ and σ_*zy*_). The cubic crystal field implies that the *x*, *y* and *z* directions are equivalent by symmetry; however, if the external magnetic field is aligned to the *x* axis (and consequently the magnetization), it breaks the equivalency. For this reason, σ_*xx*_ will be different from σ_*zz*_ and σ_*yy*_. In addition, the magnetization along *x* induces off-diagonal terms σ_*yz*_ (σ_*zy*_) leading to a scenario where an electric field in the *y*(*z*) direction can produce an excitation in the *z*(*y*) direction. The off-diagonal terms cannot be directly measured; however, they can be reconstructed from linear combinations of XAS measurements. We first focus on reconstructing the terms σ_*xx*_, σ_*yy*_, σ_*xy*_ and σ_*yx*_; hence four independent XAS measurements were performed for this purpose as shown in Table 1 ▸.

**Figure 1:**
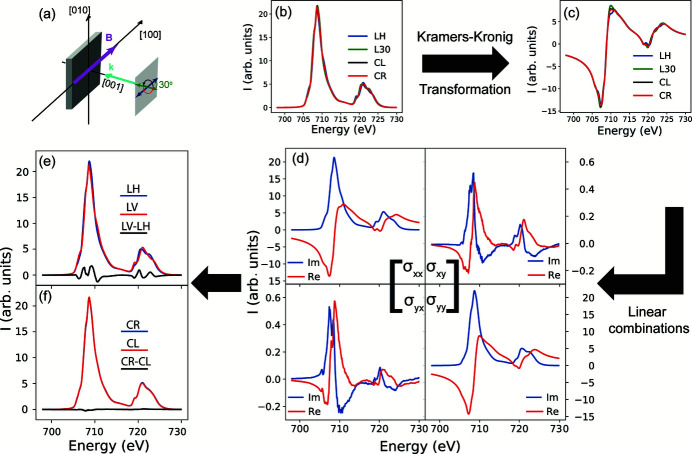
(*a*) Schematic of the scattering geometry. (*b*) Fe *L*_2,3_ XAS measurements in Fe_3_O_4_ with four linearly independent incident polarizations as described in Table 1 ▸ with the magnetic field (*B*) kept parallel to [1,0,0]. (*c*) The real part of the response function computed by applying a Kramers–Kronig transformation on the XAS measurements in (*b*). (*d*) Matrix elements of the conductivity tensor created from linear combinations of the experimental measurements while the XMLD and XMCD measurements are shown in panels (*e*) and (*f*), respectively.

The response function is a complex quantity and one needs to compute the real part of the function. The real and the imaginary parts of the response function are related to each other through the Kramers–Kronig relation, which allows the computation of the real part from the XAS measurements [see Figs. 1 ▸(*b*) and 1(*c*)]. Linear combinations of these measurements can now be created according to Table 1 ▸ to give the matrix elements of the conductivity tensor. The four matrix elements (σ_*xx*_, σ_*yy*_, σ_*xy*_ and σ_*yx*_) are shown in Fig. 1 ▸(*d*). One notices that σ_*xx*_, σ_*yy*_ are different which results in a significant XMLD [see Fig. 1 ▸(*e*)]. The off-diagonal terms σ_*xy*_ and σ_*yx*_ are about 50 times smaller than the diagonal terms and are roughly equal. These symmetric off-diagonal contributions are possibly due to the presence of small non-cubic distortion in the thin-film. In contrast to the XMLD, the XMCD is negligible as can be seen in Fig. 1 ▸(*f*).

**Figure 2:**
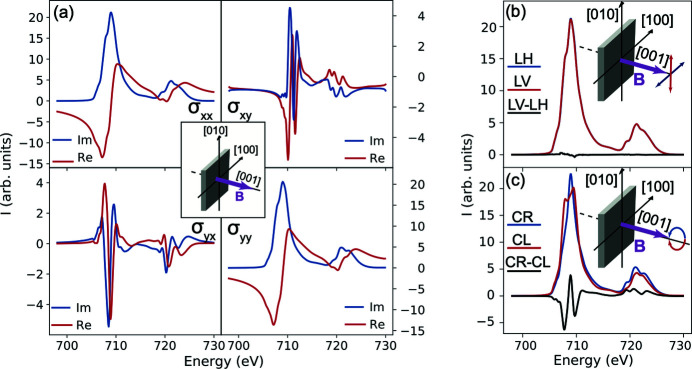
(*a*) Matrix elements of the conductivity tensor constructed for the Fe *L*_2,3_-edge in Fe_3_O_4_ with *B* || [0,0,1]. The XMLD and XMCD measurements in this configuration are shown in panels (*b*) and (*c*), respectively.

The same procedure can be used to reconstruct the conductivity tensor with the magnetic field aligned to the [0,0,1] direction from four XAS measurements. These matrix elements are shown in Fig. 2 ▸. A striking difference can be observed in comparison with Fig. 1 ▸(*d*): the off-diagonal terms σ_*xy*_ and σ_*yx*_ are nearly an order of magnitude stronger and are antisymmetric where σ_*xy*_ ≃ −σ_*yx*_. The off-diagonal term differences seen are likely due to small misalignments in the orientation of the magnetization in particular given that the [0,0,1] direction is a magnetically hard direction and does not saturate with 1 T (see Fig. 13 of Appendix *A* [App appa]) in combination with the presence of small non-cubic crystal distortion in the thin-film as we showed in earlier work (Elnaggar *et al.*, 2020 ▸). The antisymmetric off-diagonal elements result in a significant XMCD as seen in Fig. 2 ▸(*c*). On the other hand, the XMLD signal is negligible because σ_*xx*_ ≃ σ_*yy*_ [refer to Fig. 2 ▸(*b*)].

**Figure 3:**
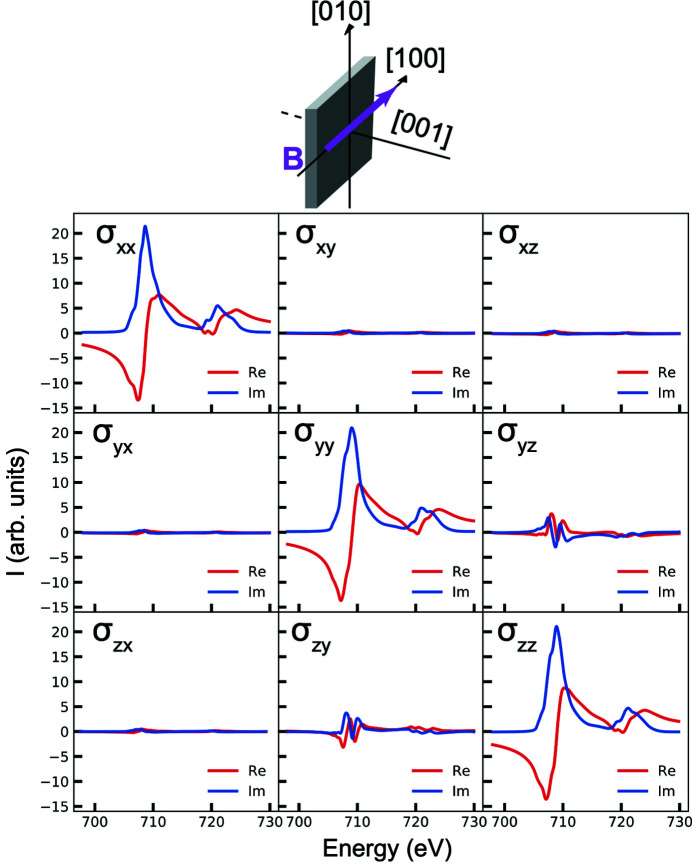
Fe *L*_2,3_ full conductivity tensor constructed for *B* || [1,0,0] in Fe_3_O_4_.

**Figure 4:**
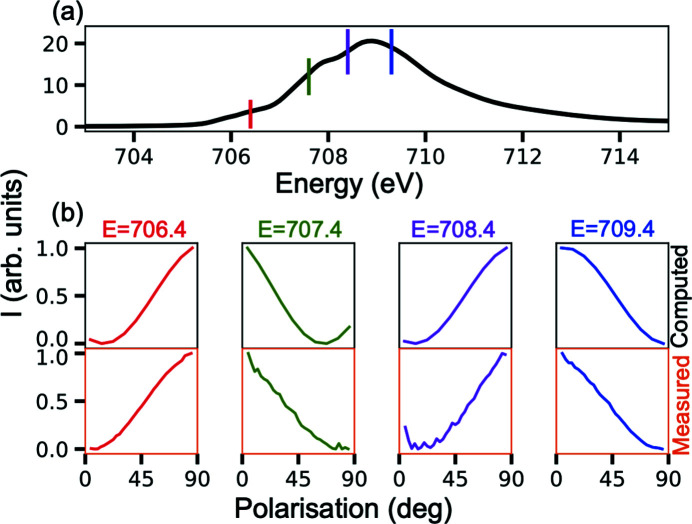
Fe *L*_3_-edge in Fe_3_O_4_. (*a*) Isotropic XAS constructed from the conductivity tensor. (*b*) Polarization dependence at *E* = 706.4 eV (red), 707.4 eV (green), 708.4 eV (magenta) and 709.4 eV (blue) where the polarization is rotated from [1,0,0] to [0,1,0]. The top row is computed from the conductivity tensor and the bottom row is experimentally measured.

The full conductivity tensor can be created by merging together the matrix elements obtained with *B* || [1,0,0] and *B* || [0,0,1] as shown in Fig. 3 ▸. This procedure assumes that the crystal field is cubic, which is an acceptable assumption given that the off-diagonal matrix elements related to the non-cubic crystal field are very small. With the full conductivity tensor at hand, we can compute XAS for any arbitrary polarization using equation (6). As such, we consider the polarization dependence as it is rotated from [1,0,0] to [0,1,0] in Fig. 4 ▸. The computed isotropic XAS and the polarization dependence at *E* = 706.4 eV (red), 707.4 eV (green), 708.4 eV (magenta) and 709.4 eV (blue) are shown in Figs. 4 ▸(*a*) and 4(*b*), respectively. The measured polarization dependences at these energies are shown in the bottom row of Fig. 4 ▸(*b*) and agree very well with the computation using the full conductivity tensor.

### 3.2. Magnetic field dependence   

The conductivity tensor shown in Fig. 3 ▸ gives the response function of the system at a certain magnetic field direction. It is of interest to find the full magneto-optical response of the system as it provides information about the anisotropic magnetic spin–orbit interaction and magnetic moments (van der Laan, 1998 ▸; Dhesi *et al.*, 2001 ▸, 2002 ▸). Symmetry operations of the crystal can be used to relate the conductivity tensor with different magnetic field directions. For example, in a cubic crystal system the conductivity tensors with the magnetic field along *x*, *y* and *z* transform into each other through a 90° rotation. Similar symmetry arguments can be used to relate the conductivity tensor as a function of the magnetic field for different crystal symmetries. Haverkort *et al.* showed that the conductivity tensor can be expressed as a sum of linear independent spectra multiplied by functions depending on the local magnetization direction as given in equation (9) (Haverkort *et al.*, 2010 ▸),
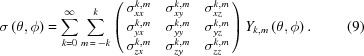
Here θ and ϕ define the direction of the local moment with θ being the polar angle, and ϕ being the azimuthal angle. *Y*_*k*,*m*_(θ, ϕ) is a spherical harmonic function and σ_*i*,*j*_ is the *i,j* component of the conductivity tensor on a basis of linear polarized light in the coordinate system of the crystal. This expression allows one to describe the full, magnetic field directional dependent, magneto-optical response of a system by using only a few linear independent fundamental spectral functions. This expression may be simplified for certain crystal systems as we will discuss in the following.

#### 3.2.1. Spherical field expansion   

**Figure 5:**
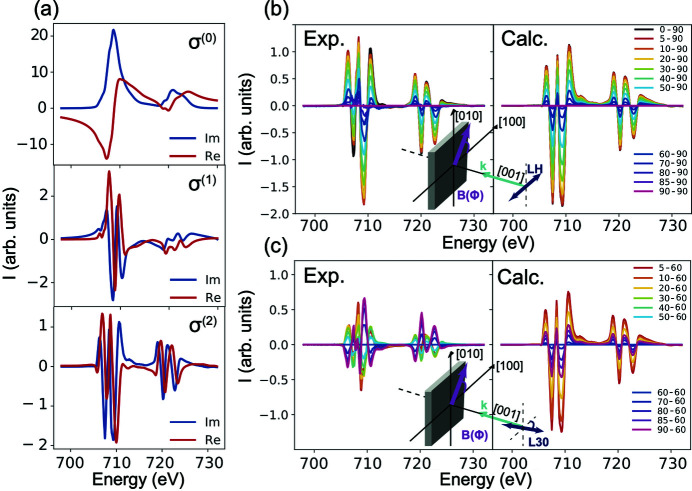
(*a*) The fundamental spectra σ^(0)^, σ^(1)^ and σ^(2)^ of Fe_3_O_4_ obtained from a spherical field expansion. (*b*) Fe *L*_2,3_ XMLD with linear horizontal polarization (LH) where the XMLD signal is defined as: XMLD = XAS(ϕ) − XAS(90°) and ϕ is the angle of the magnetic field. The measured (Exp.) and the calculated (Calc.) result from the spherical field expansion are shown in the left and right panels, respectively. (*c*) Fe *L*_2,3_ XMLD measured with linear polarization rotated 30° from the [1,0,0] (labelled L30) where the XMLD signal is defined as: XMLD = XAS(ϕ) − XAS(60°).

The crystal field splitting can in some systems be small (in comparison with other interactions such as spin–orbit coupling in rare-earth compounds) and the crystal symmetry can be considered to be nearly spherical. This approximation implies that the spectral shape modification is solely determined by the relative orientation between the magnetization and the polarization. Three fundamental spectra (σ^(0)^, σ^(1)^ and σ^(2)^) connected to the spherical harmonics *Y*_0,0_(θ,ϕ), *Y*_1,0_(θ,ϕ) and *Y*_2,0_(θ,ϕ) are required to describe the conductivity tensor with an arbitrary magnetization direction [equation (10)],
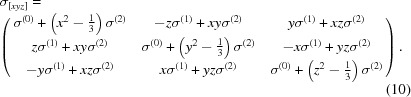
The three fundamental spectra of the spherical expansion, σ^(0)^, σ^(1)^ and σ^(2)^, in Fe_3_O_4_ are shown in Fig. 5 ▸(*a*) and can be used to compute XAS spectra for any orientation of the magnetization. To evaluate the quality of the expansion, we start by comparing the measured and the computed magnetic field angular dependence of XMLD [*I*_XMLD_ = *I*(ϕ) − *I*(90°)] with linear horizontal polarization where the magnetic field is rotated from [1,0,0] to [0,0,1] [see Fig. 5 ▸(*b*)]. The expansion reproduces the measured angular dependence well and only minor discrepancies in the absolute intensities are observed. The angular dependence in this case is given by equation (11) where the two fundamental spectra σ^(0)^ and σ^(2)^ come into play,
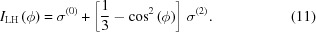
A more interesting case can be observed when the magnetic field angular dependence is measured with the polarization rotated 30° clockwise from the [1,0,0] direction {*i.e.* ε || [cos(30°),− sin(30°), 0]} as shown in Fig. 5 ▸(*c*). Contrary to the results with linear horizontally polarized light, a strong deviation from spherical symmetry is now observed. The expected angular dependence from the spherical field expansion should follow equation (12); however, the spherical field expansion completely breaks down when the polarization is aligned to a low symmetry direction. This is not a surprising result for Fe_3_O_4_ as its crystal structure is cubic and the crystal field splitting between the *t*_2*g*_ and *e*_*g*_ orbitals parametrized through 10*D*_*q*_ is ∼1 eV for Fe in Fe_3_O_4_ while the spin–orbit coupling is ∼0.05 eV and the mean field exchange interaction is ∼0.09 eV. These values suggest that the crystal field cannot be neglected, and a spherical field expansion consequently cannot describe the magneto-optical response of Fe in Fe_3_O_4_ well,
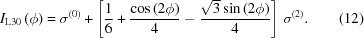



#### 3.2.2. Cubic field expansion   

**Figure 6:**
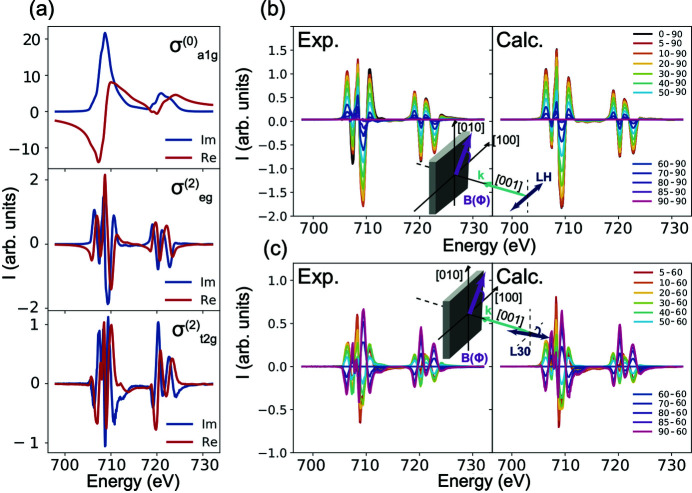
(*a*) Branching of the fundamental spectra σ^(0)^ and σ^(2)^ into 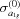, 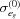 and 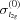 of Fe_3_O_4_ using a cubic expansion. (*b*) Fe *L*_2,3_ XMLD with linear horizontal polarization (labelled LH) where the XMLD signal is defined as: XMLD = XAS(ϕ) − XAS(90°) and ϕ is the angle of the magnetic field. The measured (Exp.) and the calculated (Calc.) result from the cubic field expansion are shown in the left and right panels, respectively. (*c*) Fe *L*_2,3_ XMLD measured with linear polarization rotated 30° from the [1,0,0] (labelled L30) where the XMLD signal is defined as XMLD = XAS(ϕ) − XAS(60°).

The local symmetry of the Fe in Fe_3_O_4_ is nearly cubic (Bragg, 1915 ▸), and therefore a more realistic treatment would be to perform a cubic field expansion. In this case, distinctly different measurements can be taken, for example along the fourfold and the threefold symmetry axes, and the fundamental spectra of order *k* branch according to their symmetry representations in the cubic point group. This is shown in equation (13) where σ^(2)^ branches to 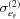 for diagonal elements and 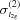 for the off-diagonal elements [see Fig. 6 ▸(*a*)]. Furthermore, higher-order *k* terms such as 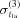 become important (Haverkort *et al.*, 2010 ▸),
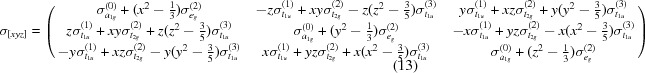
A comparison between the measured and computed magnetic field angular dependence of XMLD with linear horizontal polarization can be seen in Fig. 6 ▸(*b*). The agreement between the measurements and the computed field dependence for the spherical and cubic field expansions are of similar quality. The field dependence in this case is given by equation (14) which is of the same form as the spherical field expansion [compare equation (14) with equation (11)] and the fundamental spectra involved are very similar [compare Fig. 6 ▸(*a*) with Fig. 5 ▸(*a*)],
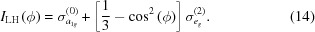
However, contrary to the spherical expansion results, an excellent agreement between the cubic field expansion and the magnetic field angular dependence performed with rotated polarization is now observed in Fig. 6 ▸(*c*). The reason for this improvement is the branching of the σ^(2)^ fundamental spectrum into 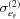 and 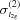 which is probed when the polarization is aligned to a low symmetry direction bringing off-diagonal elements into play. The field dependence for the 30° rotated polarization is given by equation (15) which highlights the role of the 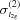 fundamental spectrum. Another important conclusion is that it is essential to measure XAS with the polarization aligned to a low symmetry direction to sensitively probe the crystal symmetry,
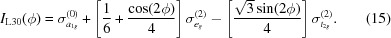



#### 3.2.3. Convergence of the field expansion   

In symmetries lower than spherical, the expansion of the spin (or the magnetic field) direction on spherical harmonics does not truncate at finite *k*. There is thus, in principle, an infinite number of linearly independent fundamental spectra. Not all of them are important and most of them will be of very low intensity. We have included in our previous analysis terms up to *k* = 3. The cubic field expansion showed a satisfactory agreement with the experimental data and only small discrepancies were showed. Here we investigate theoretically the origin of these discrepancies and the convergence of the field expansion. The quality of an expansion on the spin (or magnetic field direction) is foreseen to depend on the details of the ground state. This is because the magnetization direction depends on both the orbital and spin moments and hence whether the valence orbital moment is quenched or not will affect the efficiency of the expansion. Fe_3_O_4_ contains both types of ions, Fe^3+^ and Fe^2+^, providing us with an excellent opportunity to test the effect of the ground state for the two cases. We approach this by calculating the field dependence of XMLD in two ways:

(1) Performing a new full XMLD calculation for every magnetic field orientation.

(2) Computing once the conductivity tensor in equation (13) and then generating the field dependence of XMLD from the cubic fundamental spectra.

**Figure 7:**
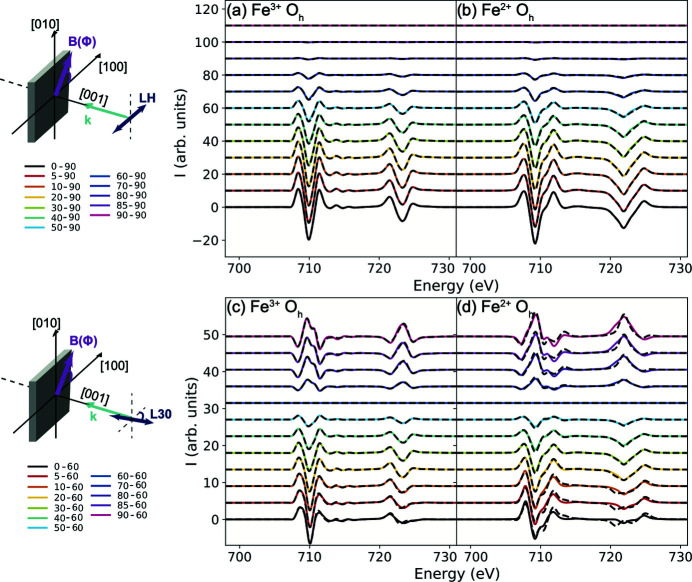
Computed *L*_2,3_ XMLD: (*a*) and (*b*) with linear polarization || [1,0,0] (labelled LH) for Fe^3+^ and Fe^2+^ in *O*_*h*_ symmetry, respectively. The XMLD is defined as: XMLD = XAS(ϕ) − XAS(90°) where ϕ is the angle of the magnetic field. The solid lines show XMLD computed individually for every magnetic field orientation while the dashed lines are computed using the cubic field expansion. Panels (*c*) and (*d*) are the results with linear polarization rotated 30° from the [1,0,0] (labelled L30) where the XMLD is defined as: XMLD = XAS(ϕ) − XAS(60°).

The first method is exact and involves no approximations. On the other hand, the accuracy of the second method depends on the order of the expansion used in the calculation. Let us first consider the magnetic field (*B*) angular dependence probed with the linear polarized X-rays aligned to [1,0,0] where *B* is rotated about [0,0,1] at ϕ = 0°. The XMLD signal [computed as XMLD = XAS(ϕ) − XAS(90°)] for Fe^3+^ and Fe^2+^ in *O*_*h*_ symmetry is shown in Figs. 7 ▸(*a*) and 7(*b*), respectively. The exact calculations (solid lines) and the cubic field expansion (dashed lines) match well which can initially suggest that the series expanded up to *k* = 3 is sufficient to describe XMLD in 3*d* transition metal oxides. Similar conclusions were reached by Arenholz *et al.* (2006 ▸, 2007 ▸) and van der Laan *et al.* (2008 ▸, 2011 ▸). However, a difference between the convergence of the series for both ions can be seen when the polarization is aligned parallel to [cos(30°),−sin(30°),0] [Figs. 7 ▸(*c*) and 7(*d*)]. Only minor discrepancies are observed for Fe^3+^ while a larger disagreement is observed for Fe^2+^.

**Figure 8:**
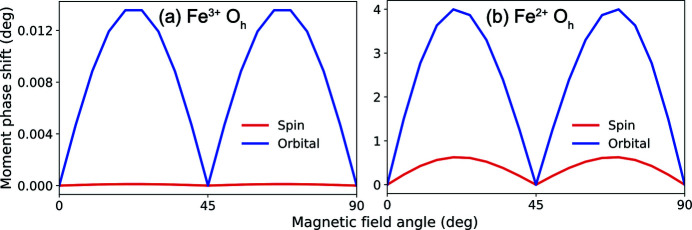
The angle between the spin/orbital (red/blue) moment and the magnetic field as a function of the magnetic field rotation angle for (*a*) Fe^3+^ in *O*_*h*_ symmetry and (*b*) Fe^2+^ in *O*_*h*_ symmetry. The magnetic field is rotated about the [0,0,1] starting from the [1,0,0] direction.

**Figure 9:**
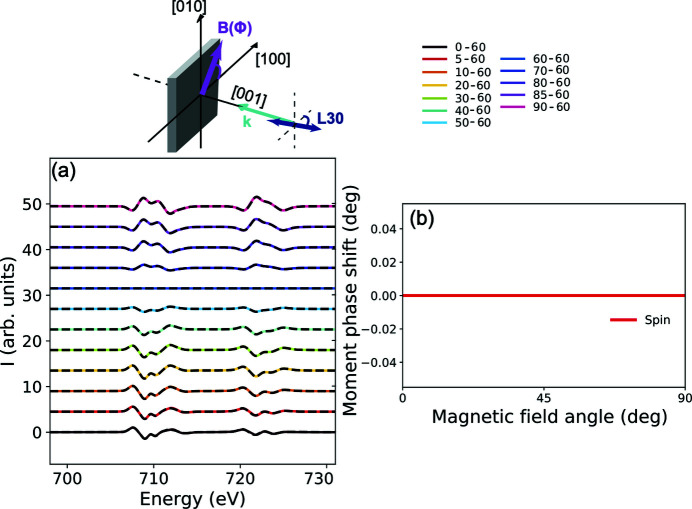
Calculations for Fe^2+^ in *O*_*h*_ symmetry with fully quenched spin–orbit coupling. (*a*) *L*_2,3_ XMLD with linear polarization rotated 30° from the [1,0,0] (labelled L30). The XMLD signal is defined as: XMLD = XAS(ϕ) − XAS(60°) where ϕ is the angle of the magnetic field. The solid lines show XMLD computed individually for every magnetic field orientation while the dashed lines are computed using the cubic field expansion [equation (15)]. (*b*) The angle between the spin moment and the magnetic field as a function of the magnetic field rotation angle.

**Figure 10:**
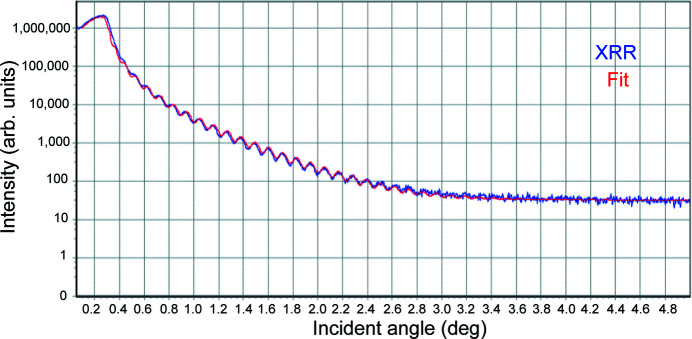
XRR measurement performed on the Fe_3_O_4_ (001)/SrTiO_3_ thin-film.

**Figure 11:**
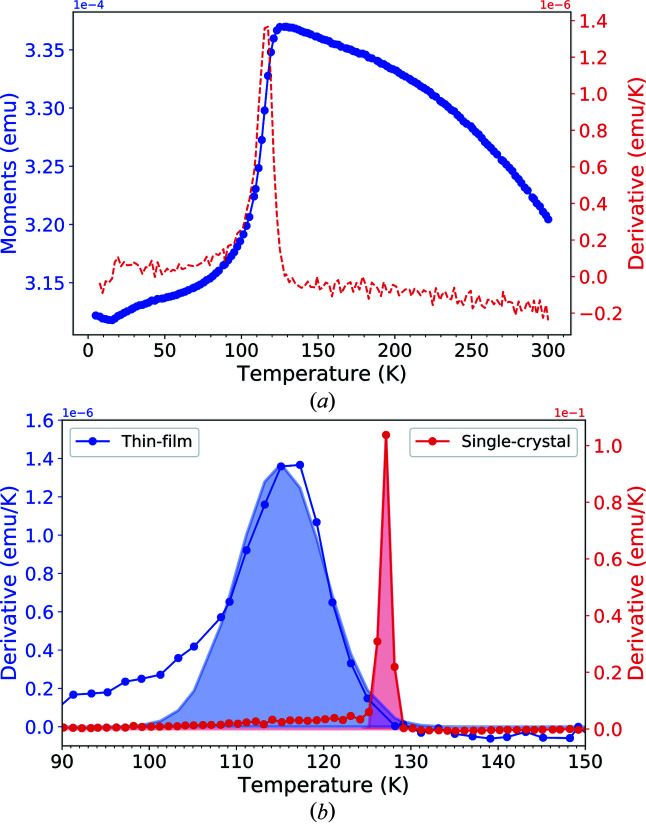
Zero-field cooled magnetization measurement (*a*) for the Fe_3_O_4_ (001)/SrTiO_3_ thin-film. (*b*) Comparison between the derivative of the magnetization measurement for the Fe_3_O_4_ (001)/SrTiO_3_ thin-film in blue and a single crystal of Fe_3_O_4_ (001) in red. Gaussian fits of the peaks for two samples are shown in filled colours.

**Figure 12:**
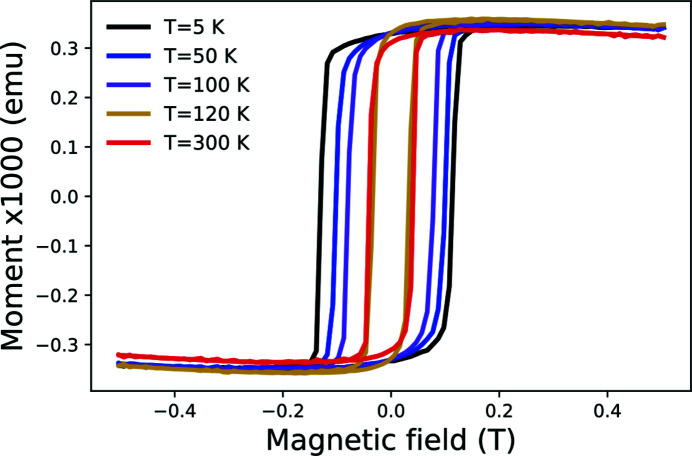
Magnetization loops measurement performed on the Fe_3_O_4_ (001)/SrTiO_3_ thin-film along the [1,0,0] axis. Five temperatures were investigated, namely *T* = 5 K, 50 K, 100 K, 150 K and 300 K.

**Figure 13:**
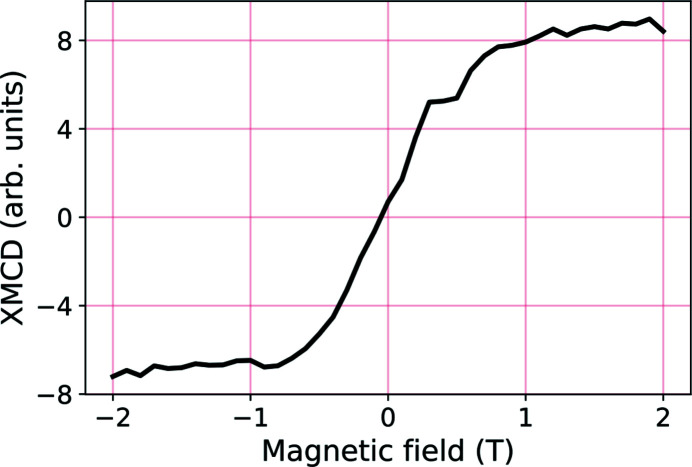
Magnetization of the Fe_3_O_4_ (001)/SrTiO_3_ thin-film measured at 200 K by recording the intensity of the XMCD at 709.5 eV as a function of the external magnetic field along the [0,0,1] direction.

The reason behind the mismatch observed lies in the ground state of Fe^2+^. In the absence of a magnetic/exchange field, the ground state of the Fe^2+^ ion in *O*_*h*_ symmetry is ^5^ *T*_2*g*_ composed of 15-fold degenerate states. This degeneracy is split by exchange and spin–orbit interactions leading to a ground state characterized by the spin and orbital momenta projections *s*_*z*_ = 1.971 (±0.01) and *l*_*z*_ = 0.98 (±0.20). On the other hand, the ground state of Fe^3+^ is characterized by the spin and orbital projections *s*_*z*_ = 2.498 (±0.001) and *l*_*z*_ = 0.001 (±0.001). These values are obtained using the wavefunction calculated by solving equation (1). The reported errors are obtained from the errors in the distortion parameters that are obtained by fitting the XMCD signal using our calculations. We note that the ground state of Fe^3+^ in *T*_*d*_ symmetry is almost identical to that in *O*_*h*_ symmetry and therefore we focus here on the *O*_*h*_ sites (refer to the Appendix *C* [App appc] for more details). As the magnetic field is rotated, the spin moment follows the field for the Fe^3+^ as shown in Fig. 8 ▸(*a*). In the case of Fe^2+^, however, the coupling between the orbital and spin momenta results in a scenario where neither the spin nor the orbital moments follow the rotation of the magnetic field [see Fig. 8 ▸(*b*)] due to the magnetocrystalline anisotropy (Alders *et al.*, 2001 ▸). The spin moment can be phase shifted from the direction of the magnetic field with ∼0.5° while the orbital moment can lag ∼4° in some directions. This causes the series to converge slower and hence higher orders of *k* are required. This is further confirmed by the calculation in Fig. 9 ▸(*a*) where the valence spin–orbit coupling is artificially switched off for Fe^2+^. Now the cubic field expansion reproduces the XMLD exquisitely well and no phase shift is observed [Fig. 9 ▸(*b*)].

## 4. Conclusions   

In conclusion, we illustrated the procedure to build the conductivity tensor from experimental measurements which describes the full magneto-optical response of the system. Applied to the Fe *L*_2,3_-edge of a 38.85 nm Fe_3_O_4_/SrTiO_3_ (001) thin-film, we showed that the convergence of the cubic expansion depends on the details of the ground state. The key aspect that affects the convergence of the expansion in this work is the valence state spin–orbit interaction. While the cubic expansion explains the angular dependence of the XMLD of Fe^3+^ with terms up to the third order, higher-order terms are required for Fe^2+^. This conclusion is expected to apply for other systems where the valence orbital moments are not quenched.

## Figures and Tables

**Figure 14:**
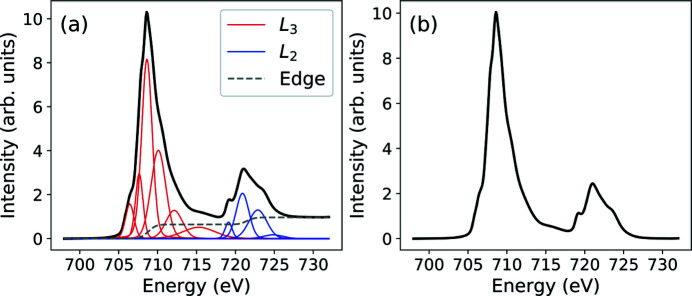
(*a*) Fitting of the XAS signal of the Fe_3_O_4_ (001)/SrTiO_3_ thin-film. (*b*) Background removal.

**Figure 15:**
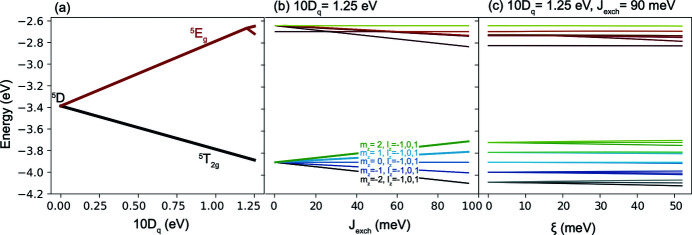
Energy diagrams illustrating the splitting of the ground state of a *d* ^6^ Fe^2+^ ion. (*a*) As a function of the octahedral crystal field 10*D*_*q*_ parameter with the spin–orbit coupling (ξ) and magnetic exchange interaction (*J*_exch_) set to zero. (*b*) As a function of *J*_exch_ with 10*D*_*q*_ = 1.25 eV and ξ = 0. (*c*) As a function of ξ with *J*_exch_ = 90 meV and 10*D*_*q*_ = 1.25 eV.

**Figure 16:**
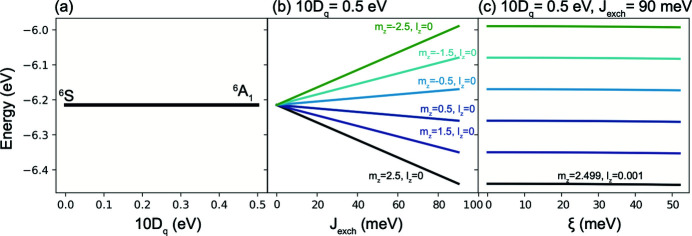
Energy diagrams illustrating the splitting of the ground state of a *d* ^5^ Fe^3+^ ion. (*a*) As a function of the tetrahedral crystal field 10*D*_*q*_ parameter (note that 10*D*_*q*_ *T*_*d*_ = −10*D*_*q*_ *O*_*h*_) with the spin–orbit coupling (ξ) and exchange interaction (*J*_exch_) set to zero. (*b*) As a function of *J*_exch_ with 10*D*_*q*_ = 0.5 eV and ξ = 0. We note that the definition of *J*_exch_ *T*_*d*_ = −*J*_exch_ *O*_*h*_ because the sites are antiferromagnetically coupled. (*c*) As a function of ξ with *J*_exch_ = 90 meV and 10*D*_*q*_ = 0.5 eV.

**Figure 17:**
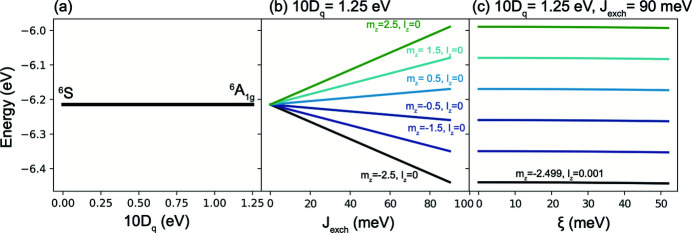
Energy diagrams illustrating the splitting of the ground state of a *d* ^5^ Fe^3+^ ion. (*a*) As a function of the octahedral crystal field 10*D*_*q*_ parameter with the spin–orbit coupling (ξ) and exchange interaction (*J*_exch_) set to zero. (*b*) As a function of with 10*D*_*q*_ = 1.25 eV and ξ = 0. (*c*) As a function of ξ with *J*_exch_ = 90 meV and 10*D*_*q*_ = 1.25 eV.

**Table 1:** Experimentally measured XAS spectra and the linear combinations required to construct σ_*xx*_, σ_*yy*_, σ_*xy*_ and σ_*yx*_ matrix elements of the conductivity tensor

Measured	Constructed
ε_LH_ 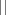 [1, 0, 0]	σ_*xx*_ = XAS_LH_
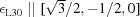	σ_*yy*_ = XAS_CL_ + XAS_CR_ − XAS_LH_
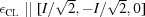	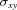 = 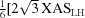 − 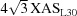 + 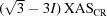 + 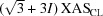 ]
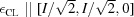	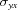 = 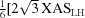 − 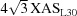 + 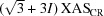 + 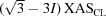 ]

**Table 2:** Gaussian peak centre and width used for fitting the *L*_3_-edge

Peak	Centre (eV)	HWFM (eV)
1	706.424	0.643
2	707.637	0.561
3	708.631	0.793
4	710.097	1.115
5	712.131	1.252
6	715.302	2.447

**Table 3:** Gaussian peak centre and width used for fitting the *L*_2_-edge

Peak	Centre (eV)	HWFM (eV)
1	719.122	0.527
2	720.908	0.963
3	722.880	1.343
4	724.852	1.422

**Table 4:** Parameters used for the XAS calculation of the Fe^2+^ *O*_*h*_ ion

Parameter	Initial state (eV)	Final state (eV)	Comment
10*Dq*	1.25	1.25	Similar to the values reported by Pattrick *et al.* (2002 ▸) and Arenholz *et al.* (2006 ▸) for Fe_3_O_4_. This crystal field parameter reproduces well XAS, XMCD and XMLD measurements in Fe_3_O_4_.
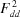	7.676	8.245	Atomic Hartree–Fock calculation scaled to 70% to take interatomic screening and mixing. This is in line with the literature such as the work by Pattrick *et al.* (2002 ▸) and Arenholz *et al.* (2006 ▸).
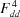	4.771	5.129	
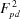	–	5.434	Atomic Hartree–Fock calculation scaled to 80%. This is in accordance to the literature such as Pattrick *et al.* (2002 ▸) and Arenholz *et al.* (2006 ▸).
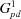	–	3.208	
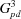	–	2.274	
ξ_*d*_	0.052	0.052	Atomic value which is a reasonable approximation as the spin–orbit coupling is nearly an atomic quantity that is material independent.
ξ_*p*_	–	8.2	
*J*_exch_	0.09	0.09	This value is based on previous 2*p*3*d* RIXS measurements that showed that the spin-flip excitation is observed at this energy [see, for example Huang *et al.* (2017 ▸) and Elnaggar *et al.* (2019*a* ▸,*b* ▸)].
Lifetime broadening (HWHM)	*L*_3_: 0.2, *L*_2_: 0.5		The lifetime broadening for the *L*_3_ used is 0.2 eV and for *L*_2_ is 0.5 eV.

**Table 5:** Parameters used for the XAS calculation of the Fe^3+^ *O*_*h*_ ion

Parameter	Initial state (eV)	Final state (eV)	Comment
10*Dq*	1.25	1.25	Similar to the values reported by Pattrick *et al.* (2002 ▸), Liu *et al.* (2017 ▸) and Arenholz *et al.* (2006 ▸) for Fe_3_O_4_. This crystal field parameter reproduces well XAS, XMCD and XMLD measurements in Fe_3_O_4_.
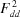	8.429	8.972	Atomic Hartree–Fock calculation scaled to 70% to take interatomic screening and mixing. This is in line with the literature such as the work by Pattrick *et al.* (2002 ▸) and Arenholz *et al.* (2006 ▸).
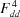	5.274	5.616	
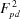	–	5.956	Atomic Hartree–Fock calculation scaled to 80%. This is in accordance with the literature such as Pattrick *et al.* (2002 ▸) and Arenholz *et al.* (2006 ▸).
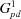	–	4.450	
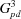	–	2.532	
ξ_*d*_	0.052	0.052	Atomic value which is a reasonable approximation as the spin–orbit coupling is nearly an atomic quantity that is material independent.
ξ_*p*_	–	8.2	
*J*_exch_	0.09	0.09	This value is based on previous 2*p*3*d* RIXS measurements that showed that the spin-flip excitation is observed at this energy [see, for example, Huang *et al.* (2017 ▸) and Elnaggar *et al.* (2019*a* ▸,*b* ▸)].
Lifetime broadening (HWHM).	*L*_3_: 0.2, *L*_2_: 0.5		The lifetime broadening for the *L*_3_ used is 0.2 eV and for *L*_2_ is 0.5 eV

**Table 6:** Parameters used for the XAS calculation of the Fe^3+^ *T*_*d*_ ion

Parameter	Initial state (eV)	Final state (eV)	Comment
10*Dq*	−0.5	−0.5	Similar to the values reported by Pattrick *et al.* (2002 ▸), Liu *et al.* (2017 ▸) and Arenholz *et al.* (2006 ▸) for Fe_3_O_4_. This crystal field parameter reproduces well XAS, XMCD and XMLD measurements in Fe_3_O_4_. We note that this is the total crystal field parameter as used in the crystal field multiplet model, *i.e.* including the effective effects of charge transfer.
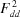	8.429	8.972	Atomic Hartree–Fock calculation scaled to 70% to take interatomic screening and mixing. This is in line with the literature such as the work by Pattrick *et al.* (2002 ▸) and Arenholz *et al.* (2006 ▸).
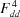	5.274	5.616	
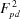	–	5.956	Atomic Hartree–Fock calculation scaled to 80%. This is in accordance with the literature such as Pattrick *et al.* (2002 ▸) and Arenholz *et al.* (2006 ▸).
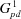	–	4.450	
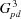	–	2.532	
ξ_*d*_	0.052	0.052	Atomic value which is a reasonable approximation as the spin–orbit coupling is nearly an atomic quantity that is material independent.
ξ_*p*_	–	8.2	
*J*_exch_	−0.09	−0.09	This value is based on previous 2*p*3*d* RIXS measurements that showed that the spin-flip excitation is observed at this energy [see, for example, Huang *et al.* (2017 ▸); Elnaggar *et al.* (2019*a* ▸,*b* ▸)].
Lifetime broadening (HWHM)	*L*_3_: 0.2, *L*_2_: 0.5		The lifetime broadening for the *L*_3_ used is 0.2 eV and for *L*_2_ is 0.5 eV.

## Appendix A Sample characterization

### A1. X-ray reflectivity measurement

The Fe_3_O_4_ (001)/SrTiO_3_ film thickness, interface and surface roughness were examined by X-ray reflectivity using a Philips XPert MRD with Cu *K*_α_ radiation (see Fig. 10 ▸). The film thickness was concluded to be ∼38.85 nm. The surface roughness was concluded to be ∼0.4 nm on average.

### A2. Magnetic measurement

Bulk magnetic properties of the Fe_3_O_4_ (001)/SrTiO_3_ thin-film were investigated using a quantum design dynacool physical properties measurement system. Magnetic moment versus temperature was measured in zero-field cooling mode with 500 Oe applied field [Fig. 11 ▸(*a*)]. A clear peak can be observed at *T* = 114.97 ± 0.29 K in the derivative signal (the Verwey transition) confirming the good stoichiometry and quality of the thin-film. A comparison between the derivative signal between the thin-film used for this work and a single crystal of the same orientation is shown in Fig. 11 ▸(*b*). The Verwey transition is significantly broader for the thin-film (FWHM = 13.03 ± 0.71 K, centre = 114.97 ± 0.29 K) in comparison with the single crystal (FWHM = 1.40 ± 0.01 K, centre = 127.08 ± 0.01 K). This could be related to defects and domain formations in the thin-film which is typical for growth on SrTiO_3_.

Hysteresis loop measurements were also performed along the [1,0,0] axis to inspect the saturation of the film below and above the Verwey transition parallel to the in-plane [1,0,0] direction (Fig. 12 ▸). The largest coercivity is observed for the lowest temperature (*H*_c_ = 0.1 T) and an external magnetic field of 0.25 T is required to saturate the in-plane magnetization. On the contrary, the magnetization is not saturated along the [0,0,1] direction with a field of *H*_c_ = 2 T as shown by the XMCD signal shown in Fig. 13 ▸.

## Appendix B Data treatment

In order to compare the XAS results measured at different magnetic field orientations it is necessary to normalize the spectra. The edge jumps (*L*_3_ and *L*_2_) were fitted by two error functions positioned at 708.8 eV and 721.5 eV, respectively [refer to the grey dashed line in Fig. 14 ▸(*a*)]. The multiplet features of the spectra were fitted by a set of Gaussian functions. Six Gaussian functions were used to fit the *L*_3_ part of the spectra [red peaks in Fig. 14 ▸(*a*)] and four to fit the *L*_2_ [blue peaks in Fig. 14 ▸(*a*)]. Only the amplitude of the functions was allowed to float between the data while the energy positions and widths were kept constant between all the data. Table 2 ▸ (Table 3 ▸) shows the centre and width of the Gaussian peaks used for the *L*_3_ (*L*_2_) edge. We normalized the spectra by setting the *L*_2_ edge jump to unity. The *L*_2,3_ edge jumps were subtracted from the data as shown in Fig. 14 ▸(*b*). Finally, the spectra were normalized to the spectral area.

## Appendix C Ground state of Fe ions in Fe_3_O_4_

### c1. Fe^2+^ in octahedral symmetry

The high spin Fe^2+^ ion in octahedral symmetry 15-fold ^5^ *T*_2*g*_ state [shown in Fig. 15 ▸(*a*)] is split by exchange interaction [90 meV applied according to equation (5)] which lowers the degeneracies leading to a triplet ground state as illustrated in Fig. 15 ▸(*b*). The spin–orbit coupling finally lifts all the degeneracies as shown in Fig. 15 ▸(*c*). The ground state is 99.8% pure in terms of crystal field configuration and has an occupation |(*t*_2*g*_)^3.9997^(*e*_*g*_)^2.0003^〉 and is 97.21% composed of the state characterized by *m*_*z*_ = −2 and *l*_*z*_ = −1. We point out that the first and second excited states are ∼22 and 53 meV higher in energy than the ground state which leads to a Boltzmann occupation of ∼75.8%, 20.6% and 3.6% of the ground, first and second excited states, respectively, at 200 K. These have been included in the calculation of the spectra. All the parameters used in the multipet calculations of Fe^2+^ XAS are reported in Table 4 ▸.

### c2. Fe^3+^ in octahedral and tetrahedral symmetry

The ground states of Fe^3+^ in *O*_*h*_ and *T*_*d*_ symmetries are almost identical with *s*_*z*_ = 2.499 and *l*_*z*_ = 0.001. Indeed, the ^6^ *A*_1_ splits to the doublet *E*_2_ and quartet *G* states; however, this splitting can be neglected (the splitting is less than 0.1 meV and is thermally populated). This is illustrated in Figs. 16 ▸ and 17 ▸ where the ^6^ *A*_1(*g*)_ state [panel (*a*)] is split mainly due to exchange interaction [panel (*b*)] while spin–orbit coupling has negligible effect [panel (*c*)] for Fe^3+^ in both *T*_*d*_ and *O*_*h*_ symmetries. We point out that the first excited state is ∼90 meV higher in energy than the ground state which leads to a Boltzmann occupation of ∼99.6% and 0.4% of the ground and first excited state, respectively, at 200 K. We therefore only used the ground state in the calculation of the spectra. All the parameters used in the multipet calculations of Fe^3+^ *O*_*h*_ and *T*_*d*_ XAS are reported in Tables 5 ▸ and 6 ▸, respectively.
